# COVID-19 Statistics in the Arab World by the End of October 2022: A Cross-Sectional Study

**DOI:** 10.7759/cureus.32670

**Published:** 2022-12-18

**Authors:** Ahmad A Alrasheedi

**Affiliations:** 1 Department of Family and Community Medicine, College of Medicine, Qassim University, Buraidah, SAU

**Keywords:** vaccine, coronavirus, covid-19, case-fatality, arab

## Abstract

Background

The coronavirus disease 2019 (COVID-19) has affected almost all world countries, including all 22 Arab countries. However, over the last 34 months, the world has suffered from the pandemic unevenly, and COVID-19 statistics are dynamic.

Objectives

The current study aimed to use COVID-19 data to examine COVID-19 statistics (including the number of cases/deaths/tests) in Arab countries by the end of October 2022 and compare the findings with global statistics. This study was also used to determine the extent to which statistics vary across Arab countries.

Methods

The primary data on COVID-19 for each Arab country were obtained from the "Worldometer" website. The data include the cumulative incidence of COVID-19 per country, the cumulative number of deaths, the total number of tests performed, the number of cases per million population, the number of deaths per million, the number of tests per million, and the total population. The case-fatality rate (CFR) was calculated (number of deaths/number of cases). In addition, the median age for each Arab country was extracted from the United Nations website. The rate of vaccination coverage (people who received two doses) was extracted from the "Our World in Data" website. COVID-19 statistics were further analyzed in Arab countries in Asia compared to those in Africa at the end of 2020, 2021, and October 2022. To compare the Arab countries to the globe, COVID-19 data for each continent were obtained. The Spearman correlation coefficient was used to determine the relationship between different variables across Arab countries.

Results

As of November 1, 2022, about 636 million COVID-19 cases and 6.6 million deaths had been recorded worldwide. Arab countries accounted for nearly 2.21% and 2.62% of all cases and deaths, respectively. In general, the mean deaths per million and the mean cases per million for Arab countries were lower than those of the world's countries, although Arab countries recorded a higher mean case-fatality rate. Alternatively, Arab countries in aggregate recorded fewer deaths per million (381) than the world (830). However, statistics across Arab countries have been inconsistent; Arab countries in Africa were less affected. Arab countries have performed approximately 359 million tests (5.29% of all tests), 93% of which were performed by Arab countries in Asia. Moreover, 54.4% of all tests were performed in the United Arab Emirates. Yemen, Somalia, Sudan, Algeria, Syria, Comoros, and Djibouti were the least affected Arab countries based on the number of deaths per million. With the exception of Comoros, these countries were among the least vaccinated in the Arab world.

Conclusions

In general, Arab countries have been less affected by the COVID-19 pandemic than the rest of the world. However, statistics vary across Arab countries, especially regarding the number of tests performed. Given the natural immunity acquired during the three years and the relatively good vaccine coverage in the Arab world, it is important to reconsider the definition of a suspected case and establish more specific criteria for testing.

## Introduction

Coronavirus disease 2019 (COVID-19) is an infectious disease caused by severe acute respiratory syndrome coronavirus-2 (SARS-CoV-2) that emerged in December 2019 [1]. The spectrum of COVID-19 symptoms is wide. Most sick patients present with respiratory tract symptoms [1]. Defining COVID-19 patients based on their clinical features is difficult because the presentations connected with respiratory tract infections commonly overlap, and their causes include a variety of viruses and bacteria, but most are viral in origin. So, as with other viruses, the diagnosis of COVID-19 cannot be established without microbiologic testing [2]. On the other hand, most people infected with SARS-CoV-2 who fall sick experience mild symptoms and recover spontaneously [3]. Furthermore, asymptomatic SARS-CoV-2 infections are common [4]. Therefore, a confirmed case of COVID-19 does not require the criteria for a suspect case to be met but simply a positive nucleic acid amplification test (most commonly a reverse-transcription polymerase chain reaction (RT-PCR) assay) [5].

The disease appeared first in Wuhan, China. Thereafter, cases increased dramatically until they reached nearly 80,000, with around 3,000 deaths attributed to COVID-19 by the end of February 2020 [6]. From about mid-February, as China seemed to be on its way to containing the disease, cases began appearing in other parts of the world. Specifically, Spain, Italy, and Iran suffered first, followed by many other countries [6,7]. So, on March 11, 2020, the World Health Organization (WHO) declared COVID-19 a pandemic [1].

Over the last 34 months, the world has suffered from the pandemic unevenly. COVID-19 statistics have not been consistent across the world and may change dramatically over time [8]. Contrary to initial expectations, Africa was the least affected continent by the COVID-19 pandemic [8,9]. The most important reason for this variability is the difference between countries in terms of the definition of suspected cases, testing strategies, criteria for when a result is considered positive, capacity to perform testing, and reporting methods [8,9]. Moreover, statistics may change significantly even within the same country [8]. To illustrate, at the end of 2020, Vietnam had only 35 deaths attributed to COVID-19, but the number rose in 2021 to 32,394; the number multiplied 925 times [9]. Additionally, demographic factors, such as the difference in median age, may influence the COVID-19 pandemic's course [8], as infection with SARS-CoV-2 causes greater mortality in older than younger adults [7].

The Arab world is a geopolitical term for a geographical area with a common history, language, and culture. The Arab world consists of 22 countries that are members of the Arab League. They are distributed as 12 in Asia (West Asia) and 10 in Africa (North Africa, East Africa, and West Africa). The Arab world stretches across more than nearly 13.4 million square kilometers, representing 10% of the world's land [10]. As of July 1, 2020, the Arab countries' population (including non-Arab nationals) was around 440 million [6]. The countries in the Arab world are diverse in terms of economics, population density, government systems, and healthcare services. Outside of the high-income oil countries of the Arabian Gulf (also known as countries of the Gulf Cooperation Council), countries have not been able to maintain functioning public health systems for their citizens [11]. Furthermore, some Arab countries, such as Yemen and Somalia, have poor governance and poor health indicators [11]. Moreover, Arab countries vary greatly in terms of vaccination coverage [12]. Therefore, it is expected that Arab countries that have some deficiencies in the health system or political instability would have been more significantly affected by the COVID-19 pandemic.

Although many studies on the epidemiology of COVID-19 in Arab countries have been published, to the best of our knowledge, there has been little recent scientific research that has examined the epidemiology of the pandemic in the Arab world as a whole. Moreover, understanding of COVID-19 is still evolving, and COVID-19 statistics are dynamic. One earlier study aimed to assess the prevalence of COVID-19 in the Arab world from February 2020 to February 2021 [13]. So, the current study aims to use COVID-19 data to examine COVID-19 statistics in Arab countries by the end of October 2022 and compare the findings with global statistics. This study was also used to determine the extent to which statistics vary across Arab countries.

## Materials and methods

Unless otherwise specified, the primary data on COVID-19 were obtained from the "Worldometer" website [6]. Data on COVID-19 were copied for all world countries and territories at the end of 2020, 2021, and October 2022 [6]. Then, they were stored in Excel files. Data from cruise ships, such as the Diamond Princess, were excluded. The data used in this analysis consists of the cumulative incidence of COVID-19 (confirmed cases) per country, the cumulative number of deaths, the total number of tests performed, the number of cases per million population, the number of deaths per million population (D/M), the number of tests per million population, and the total population.

According to the objectives, the required data for each Arab country was obtained from the stored files. The case-fatality rate (CFR) was calculated by dividing the number of deaths by the number of confirmed cases. A CFR is generally expressed as a percentage [7,14]. Arab countries in Asia include Iraq, Jordan, Lebanon, Palestine, Syria, and Yemen, as well as the Arabian Gulf countries (Bahrain, Kuwait, Oman, Qatar, United Arab Emirates [UEA], and Saudi Arabia). Arab countries in Africa include Algeria, Comoros, Djibouti, Egypt, Libya, Mauritania, Morocco, Sudan, Somalia, and Tunisia. The period covered was from January 2020 to October 2022.

In addition, the median age for the year 2021 for each Arab country was extracted from the United Nations website [15]. The rate of vaccination coverage (people with a complete initial protocol: two doses) in Arab countries was extracted from the "Our World in Data" website [12]. To avoid using too many digits, the number of tests per population is calculated, instead of using the number of tests per million, by dividing the number of tests by the population.

To better compare COVID-19 statistics across the Arab world, Arab countries were divided into two groups based on the continent they belong to. Then, the statistics were analyzed at the end of 2020, 2021, and October 2022. Results were presented as numbers, percentages, and means as appropriate. Also, to compare the Arab countries to those around the globe, COVID-19 data for each continent were obtained. The mean CFR, mean D/M, and mean cases per million for all world countries/territories were measured.

Statistical analysis was carried out using the Statistical Package for Social Sciences (SPSS, version 26, IBM SPSS, Armonk, NY). The Spearman correlation coefficient was used to determine the relationship between different variables across Arab countries. A P-value of less than 0.05 was considered significant. Ethical approval from an Institutional Review Board was not required due to the secondary analysis of publicly available data.

## Results

By the end of 2020, Arab countries had 3,272,961 confirmed cases of COVID-19 (approximately 3.9% of cases recorded worldwide) and 56,643 deaths attributed to COVID-19 (3% of all deaths). Nearly 55 million tests were performed, representing 4.5% of the tests performed worldwide. The mean CFR across Arab countries was 3.34%, ranging from 0.17% to 29.06%. By another calculation, the CFR for Arab countries in aggregate was 1.73%. Iraq was the most affected country in terms of the number of cases and the number of deaths, while, based on the number of deaths per million, Tunisia was the worst (Table 1).

**Table 1:** COVID-19 statistics for Arab countries by the end of 2020 (sorted according to the number of deaths per million). *CFR: case-fatality rate, D/M: the number of deaths per million, SA: Saudi Arabia, UEA: United Arab Emirates. ^#^as of July 1, 2020.

	No. of cases	No. of deaths	CFR*	D/M*	No. of tests	Population^#^
Tunisia	139,140	4,676	3.36%	394	615,770	11,818,619
Jordan	294,494	3,834	1.30%	374	3,175,555	10,203,134
Iraq	595,291	12,813	2.15%	315	4,547,545	40,222,493
Oman	128,867	1,499	1.16%	290	571,472	5,106,626
Palestine	138,004	1,400	1.01%	271	884,061	5,101,414
Kuwait	150,584	934	0.62%	217	1,254,832	4,270,571
Lebanon	181,503	1,468	0.81%	216	1,988,768	6,825,445
Libya	100,277	1,478	1.47%	214	546,514	6,871,292
Bahrain	92,675	352	0.38%	203	2,366,275	1,701,575
Morocco	439,193	7,388	1.68%	199	4,457,349	36,910,560
SA*	362,741	6,223	1.72%	177	11,010,384	34,813,871
Qatar	143,834	245	0.17%	87	1,241,406	2,881,053
Mauritania	14,364	347	2.42%	74	123,133	4,649,658
Egypt	136,644	7,576	5.54%	73	1,000,000	102,334,404
UAE*	207,822	669	0.32%	67	20,890,341	9,890,402
Algeria	99,311	2,751	2.77%	62	No data	43,851,044
Djibouti	5,831	61	1.05%	61	100,294	988,000
Syria	11,434	711	6.22%	40	No data	17,500,658
Sudan	23,316	1,468	6.30%	33	No data	43,849,260
Yemen	2,099	610	29.06%	20	17,404	29,825,964
Comoros	823	10	1.22%	11	No data	869,601
Somalia	4,714	130	2.76%	8	No data	15,893,222

By the end of 2021, the number of cases and the number of deaths had nearly tripled in the Arab world compared to the end of 2020, while the number of tests had doubled 4.5 times, as shown in Table 2. Additionally, Table 2 shows COVID-19 statistics for the Arab world by continent at the end of 2020, 2021, and October 2022.

**Table 2:** COVID-19 statistics for the Arab world based on the continent by the end of 2020, 2021, and October 2022. *CFR: case-fatality rate, C/M: the number of cases per million, D/M: the number of deaths per million. ^#^The mean is the sum of all values in the dataset, divided by the total number of values. While the CFR (not mean CFR), for example, is the total number of deaths of all countries included/the total number of cases of the same countries *100.

	End of 2020			End of 2021			As of November 1, 2022		
	Africa	Asia	All	Africa	Asia	All	Africa	Asia	All
No. of cases	963,613 (29.44%)	2,309,348 (70.56%)	3,272,961	2,812,203 (40.44%)	6,954,036 (59.56%)	9,766,239	3,884,322 (27.30%)	10,198,982 (72.70%)	14,083,304
No. of deaths	25,885 (45.70%)	30,758 (54.30%)	56,643	79,999 (51.58%)	75,089 (48.42%)	155,088	91,151 (52.73%)	81,711 (47.27%)	172,862
CFR*	2.69%	1.33%	1.73%	2.84%	1.08%	1.59%	2.35%	0.80%	1.23%
Mean CFR^#^	2.86%	3.74%	3.34%	3.38%	2.88%	3.11%	2.99%	2.53%	2.74%
C/M*	3,562	13,601	7,434	10,208	40,225	21,781	13,971	58,143	31,059
Mean C/M^#^	5,227	24,451	15,712	18,182	63,887	43,112	25,255	118,863	76,314
D/M*	96	181	129	290	434	346	328	466	381
Mean D/M^#^	113	190	155	438	588	519	491	648	577
No. of tests	6,843,060 (12.49%)	47,948,043 (87.51%)	54,791,103	21,926,514 (8.87%)	225,183,298 (91.13%)	247,109,812	26,305,374 (7.32%)	332,856,972 (92.68%)	359,162,346
Tests/pop.	0.03	0.28	0.12	0.08	1.30	0.55	0.09	1.90	0.79
Population	270,502,643	169,792,102	440,294,745	275,496,490	172,877,871	448,374,361	278,026,309	175,410,811	453,437,120

In 2022, the population of Arab countries reached approximately 453 million (5.7% of the world's population), slightly larger than South America, with about 66% of the Arab countries' population living in Africa. In the first ten months of 2022, nearly 347 million cases were recorded worldwide, representing 54.65% of all cases recorded since the pandemic's beginning. As of November 1, 2022, about 636 million cases and 6.6 million deaths had been recorded worldwide, as shown in Table 3. Arab countries accounted for nearly 2.21% and 2.62% of all cases and deaths, respectively. The CFR of Arab countries combined in Africa (2.35%) was higher than that in Asia (0.80%). However, Arab countries in Africa were better based on the deaths per million and more cases per million than Arab countries in Asia. The CFR for Arab countries combined in 2022 decreased to 1.23%. About 359 million tests were performed by Arab countries, and most (93%) were done in Asia. The mean CFRs, mean cases per million, and mean deaths per million for the world's countries by the end of October 2022 were 1.32%, 183,552, and 1,208, respectively. Based on this information and by looking at Tables 2-3, a comparison of COVID-19 statistics between the Arab world and the rest of the world can be made.

**Table 3:** COVID-19 statistics among the six continents by the end of October 2022 (sorted according to the number of deaths per million). *CFR: case-fatality rate, D/M: the number of deaths per million. ^#^North America includes Mexico and Caribbean countries.

	No. of cases	No. of deaths	CFR*	D/M*	No. of tests	Tests/pop.	Population
South America	64,447,745	1,332,969	2.07%	3,045	237,653,983	0.54	437,694,443
Europe	234,475,143	1,942,549	0.83%	2,599	2,778,426,689	3.72	747,543,837
North America^#^	117,850,921	1,552,847	1.32%	2,596	1,262,347,121	2.11	598,140,916
Oceania	12,620,052	21,586	0.17%	497	88,291,944	2.03	43,469,030
Asia	193,886,632	1,487,935	0.77%	316	2,310,875,850	0.49	4,711,356,783
Africa	12,673,817	257,854	2.03%	183	108,756,579	0.08	1,406,728,744
All	635,954,310	6,595,740	1.04%	830	6,786,352,166	0.85	7,944,933,753

By the end of October 2022, Yemen, Somalia, Sudan, Algeria, and Syria were the least affected Arab countries based on the number of deaths per million, as shown in Table 4. Surprisingly, more than half of the tests were performed in one country, UEA (54.4%). In addition to Jordan, the Gulf countries were the only Arab countries that performed more tests than their populations. In other words, about 80% of tests were done in the Gulf countries. Across Arab countries, the mean vaccination coverage rate was 43.8%, while the median age is 25.4; 27.1 and 23.3 in Arab countries of Asia and Africa, respectively.

**Table 4:** COVID-19 statistics for Arab countries by the end of October 2022 (sorted according to the number of deaths per million). *CFR: case-fatality rate, D/M: the number of deaths per million, Age: median age in years, SA: Saudi Arabia, UEA: United Arab Emirates. ^#^Vaccination coverage indicates people with a complete initial protocol (two doses).

	No. of cases	No. of deaths	CFR*	D/M*	No. of tests	Age*	Vac.^#^	Population
Tunisia	1,146,593	29,259	2.55%	2,429	4,966,900	31.7	51.7%	12,046,656
Lebanon	1,218,779	10,708	0.88%	1,602	479,5578	19.8	43.9%	6,684,849
Jordan	1,746,997	14,122	0.81%	1,371	17,201,885	20.1	43.4%	10,300,869
Palestine	620,816	5,404	0.87%	1,011	3,078,533	32.7	33.9%	5,345,541
Libya	507,051	6,437	1.27%	914	2,483,005	23.9	18.1%	7,040,745
Bahrain	690,401	1,529	0.22%	857	10,494,673	32.8	83.3%	1,783,983
Oman	398,775	4,260	1.07%	800	25,000,000	29.8	66.6%	5,323,993
Iraq	2,461,484	25,358	1.03%	601	19,377,732	32.7	17.8%	42,164,965
Kuwait	662,073	2,568	0.39%	586	8,416,379	19.2	78.3%	4,380,326
Morocco	1,265,650	16,281	1.29%	431	12,666,157	26.3	62.8%	37,772,756
SA*	822,718	9,409	1.14%	262	44,617,031	33	69.6%	35,844,909
UAE*	1,037,929	2,348	0.23%	233	195,356,320	28.8	97.1%	10,081,785
Egypt	515,645	24,613	4.77%	232	3,693,367	28.8	36%	106,156,692
Qatar	469,709	684	0.15%	230	4,042,980	20.9	95.6%	2,979,915
Mauritania	63,374	997	1.57%	203	995,736	33	30.1%	4,901,981
Djibouti	15,690	189	1.20%	186	305,941	29.8	21.4%	1,016,097
Comoros	8,762	161	1.84%	177	No data	15.2	47.5%	907,419
Syria	57,362	3,163	5.51%	163	146,269	18.7	9.5%	19,364,809
Algeria	270,839	6,881	2.54%	152	230,861	20.9	14.43%	45,350,148
Sudan	63,481	4,972	7.83%	108	562,941	31.7	9.7%	45,992,020
Somalia	27,237	1,361	5%	81	400,466	32.8	33.7%	16,841,795
Yemen	11,939	2,158	18.08%	69	329,592	38.3	2%	31,154,867

On the other hand, the number of deaths per million was significantly proportional to the number of cases and the number of cases per million, as shown in Table 5. Also, the CFRs were negatively correlated with the number of cases per million, the median age, and the rate of vaccination coverage. The number of deaths was significantly proportional to the number of cases. The vaccination coverage rate was directly proportional to the number of tests, the number of cases per million, the number of tests per million, and the median age.

**Table 5:** The correlation between different variables across Arab countries. **Correlation is significant at the 0.01 level (two-tailed). *Correlation is significant at the 0.05 level (two-tailed). ^#^CFR: case-fatality rate, C/M: the number of cases per million, D/M: the number of deaths per million, vac: vaccination.

		D/M^#^	CFRs^#^	No. of deaths	No. of cases	No. of tests	C/M^#^	Tests/M	Median age	Vac.^#^
D/M^#^	Pearson correlation	1	-.309-	.537^**^	.521^*^	-.107-	.493^*^	-.051-	.321	.143
	Sig. (two-tailed)		.162	.010	.013	.644	.020	.827	.145	.524
	N	22	22	22	22	21	22	21	22	22
CFRs^#^	Pearson correlation	-.309-	1	-.084-	-.400-	-.222-	-.430-^*^	-.260-	-.478-^*^	-.565-^**^
	Sig. (two-tailed)	.162		.709	.065	.333	.046	.255	.024	.006
	N	22	22	22	22	21	22	21	22	22
No. of deaths	Pearson correlation	.537^**^	-.084-	1	.686^**^	-.089-	-.052-	-.220-	.074	-.101-
	Sig. (two-tailed)	.010	.709		.000	.702	.819	.337	.743	.655
	N	22	22	22	22	21	22	21	22	22
No. of cases	Pearson correlation	.521^*^	-.400-	.686^**^	1	.249	.362	.161	.265	.225
	Sig. (two-tailed)	.013	.065	.000		.277	.098	.485	.233	.314
	N	22	22	22	22	21	22	21	22	22
C/M^#^	Pearson correlation	.493^*^	-.430-^*^	-.052-	.362	.086	1	.322	.558^**^	.573^**^
	Sig. (two-tailed)	.020	.046	.819	.098	.711		.154	.007	.005
	N	22	22	22	22	21	22	21	22	22
Vac.^#^	Pearson correlation	.143	-.565-^**^	-.101-	.225	.511^*^	.573^**^	.603^**^	.765^**^	1
	Sig. (two-tailed)	.524	.006	.655	.314	.018	.005	.004	.000	
	N	22	22	22	22	21	22	21	22	22

## Discussion

By November 1, 2022, the number of COVID-19 tests performed worldwide had reached 6.8 billion, a relatively large number. To understand this, we should look at the testing strategy followed globally. In April 2021, the United States Centers for Disease Control and Prevention (CDC) updated testing strategies for SARS-CoV-2 [16]. Diagnostic testing should be performed on anyone who has signs and symptoms consistent with COVID-19 and/or following recent known or suspected exposure to SARS-CoV-2 [16]. However, the spectrum of COVID-19 symptoms and signs is very wide. The symptoms include almost every respiratory symptom and almost every general symptom, as well as some gastrointestinal symptoms. Specifically, the symptoms include but are not limited to, cough, fever, myalgias, headache, dyspnea, sore throat, diarrhea, nausea/vomiting, any smell and/or taste abnormality, rhinorrhea and/or nasal congestion, chills/rigors, fatigue, confusion, and chest pain or pressure [17]. These symptoms are neither specific to COVID-19 nor any other disease [2]. Many diseases (infectious and non-infectious) are similar to COVID-19, especially respiratory and heart diseases. Additionally, over 200 subtypes of viruses (including coronaviruses and influenza) have been associated with the same symptoms [18].

So, COVID-19 can only be confirmed by specific viral tests (most commonly PCR tests) [2,5]. However, some countries relied only on clinical features to diagnose COVID-19. For example, North Korea has considered the presence of a fever as a case of COVID since its first case was discovered on May 12, 2022 [19]. Algeria performed fewer tests than the number of cases detected (Table 4), which possibly means that Algeria either has used other diagnostic methods or has not updated the data as it should. Even in the same country, there could be different approaches to reporting data (such as variable frequency of reporting) about COVID-19 cases, deaths, tests, and vaccines, as in the United States [20]. Such complexities cause some confusion about the COVID-19 statistics around the world. Furthermore, there is a difference between a clinical case definition - applied to an individual presenting to health care - and a surveillance definition used to collect information for public health purposes [21]. So, many symptomatic patients were diagnosed by active surveillance programs [22]. However, the diagnostic accuracy of symptoms for COVID-19 is moderate to low, and any testing strategy using symptoms as a selection method will result in both a large number of missed cases and a large number of people needing testing [2].

On the other hand, because of the high rate of asymptomatic patients [4], screening asymptomatic people who do not have known, suspected, or reported exposure to SARS-CoV-2 is another testing strategy followed by the CDC [16]. In the current pandemic, mass screening programs were suggested by the WHO as an effective precautionary measure to control the spread of the virus [23]. So, mass testing campaigns were conducted in crowded pockets [24]. This means that the test was made available to the public, regardless of medical history or epidemiological contact. However, most world countries are unable to implement the mass screening strategy; countries differ in terms of testing capacity and testing strategies [8]. The number of cases and the number of deaths depend largely on the number of tests. In general, high-income countries tend to perform more tests. It seems that the Gulf countries have followed a similar policy; they have tested nearly five times more than their population. This is almost similar to Europe and the United States. Globally, Austria was the most testing country relative to its population (22 times/population), followed by Denmark and the UAE [6].

Among Arab countries, Yemen, Somalia, Sudan, Algeria, Syria, Djibouti, and Comoros had less than 200 deaths per million, a rate much lower than the mean (577). In addition to Egypt and Mauritania, these countries also detected fewer than 20,000 cases per million, a rate much lower than the mean (76,314). The total number of tests these countries conduct represents 0.17% of the tests performed in the Arab world. Although Egypt is the most populous Arab country, it has been affected less than most other Arab countries. However, it had a high CFR, which is the same for most countries performing fewer tests, but as many studies point out, the CFR, unlike the mortality rate, provides a short view because it does not take into account the entire population [7,8,14]. Egypt has tested the equivalent of 3% of the population. An Egyptian study suggested that the low COVID-19 prevalence in Egypt does not reflect reality, as many infected people pass without any laboratory confirmation [25]. In contrast, Saudi Arabia, the best Arab country in terms of gross domestic product [26], performed more tests than its population, resulting in more cases being discovered and thus lowering the CFR value. Saudi Arabia followed a mass screening strategy [23]. Tunisia, the most affected among the Arab countries, together with South Africa accounted for more than half of the deaths in the African continent, although both countries represent approximately 5% of the continent's population [8].

For global comparison, the mean deaths per million and the mean cases per million for Arab countries were less than those of the world's countries, although they recorded a higher mean CFR. By another calculation, Arab countries in aggregate were also better based on deaths per million and cases per million, even though they had a higher bit CFR (Tables 3-4). The WHO has recommended using certain criteria (any acute respiratory illness and history of contact with a confirmed or probable COVID-19 case in the 14 days prior to the onset of symptoms) to identify a suspected case of COVID-19 since the very beginning of the outbreak [27]. However, although most criteria are modifications of the WHO recommendations, the specific clinical features and epidemiological risks for triggering the evaluation of patients with suspected COVID-19 differ widely among countries. The rationale for these differences may be related to each country’s resources, politics, experience with previous epidemics, health insurance systems, and other unknown factors [27]. There is no consensus regarding the best diagnostic criteria for identifying a suspected case of COVID-19 [27]. Moreover, the criteria could significantly change over time, resulting in changes in statistics.

By November 2022, the Gulf countries topped the list of Arab countries in terms of vaccination coverage, followed by Tunisia and Morocco, where more than half of the population had been fully vaccinated. While less than 20% of the population of Libya, Iraq, Algeria, Sudan, Syria, and Yemen has been fully vaccinated. About 62.75% of the world's population has completed the initial protocol of the COVID-19 vaccine [12]. Only 19.25% of people in low-income countries have received two doses [12]. Arab countries with the lowest vaccination coverage were almost the least affected by the pandemic. Statistically, vaccination coverage was negatively associated with CFRs and positively associated with the number of tests, the number of tests per million, and the number of cases per million. Countries with greater vaccination coverage seem to have active COVID-19 testing programs and therefore more cases. A previous study including 191 countries around the world showed that vaccination is directly proportional to the number of cases detected and negatively related to CFRs [28]. However, these observations suggest an association, not necessarily a causal relationship. Moreover, measuring the effects of vaccines using population data is not straightforward. It may produce misleading results and inherent biases that are difficult to pin down, particularly if an important confounder such as the seasonal effect is overlooked, as well as the great difference between countries in statistics. It is also important to consider the natural immunity acquired over time.

Since the emergence of the COVID-19 pandemic, 2021 has been the worst year globally in terms of the number of deaths. While the first ten months of 2022 were the best although a huge number of cases (which represents the number of positive tests) were recorded during this period. One recent narrative review highlighted that most people suffering from COVID-19 develop a natural immunity of both cell-mediated and humoral type, which is effective over time and provides protection against both reinfection and serious illness, while vaccine-induced immunity decays faster than natural immunity [29]. Natural immunity after COVID-19 seems comparable or superior to the one induced by anti-SARS-CoV-2 vaccination [29]. This could provide a reasonable explanation as to why the burden of COVID-19 is low in some Arabic countries, such as Yemen, Somalia, and Sudan. Given the high rate of asymptomatic SARS-CoV-2-infected individuals [4], it is estimated that the rate of prior exposure to SARS-CoV-2 exceeds the incidence of reported cases by about tenfold or more [30]. So, it is estimated that at least 6.6 billion infections with SARS-CoV-2 have occurred.

Finally, this study provides an updated overview of COVID-19 statistics around the Arab world. However, there are some limitations to this study. The most important limitation is that the quality of the obtained information is dependent on the quality of the raw data. Not all countries were reporting COVID-19 statistics at the same frequency and quality. Moreover, COVID-19 data were obtained from one source.

## Conclusions

In general, Arab countries have been less affected by the COVID-19 pandemic than the rest of the world. However, statistics across Arab countries have been inconsistent; Arab countries in Africa were less affected. Arab countries have performed approximately 359 million tests (5.29% of the tests conducted globally), approximately 93% of which were performed in Asia. Specifically, 80% of the 359 million tests have been done in the Gulf countries. Given the natural immunity acquired during the three years and the relatively good vaccine coverage in the Arab world, it is important to reconsider the definition of a suspected case and establish more specific criteria for testing, especially for those countries that have performed excessive tests.
